# Comparative evaluation of YOLO models for retained root fragment detection

**DOI:** 10.1371/journal.pone.0358937

**Published:** 2026-10-23

**Authors:** Chunyan Ma, Lei He, Kang Xia, Xiaoyuan Zheng, Haibing Yang

**Affiliations:** 1 Department of Stomatology, The Changzhou No. 2 People’s Hospital, The Third Affiliated Hospital of Nanjing Medical University, Changzhou Medical Center of Nanjing Medical University, Changzhou, Jiangsu, China; 2 Department of Information, The Changzhou No. 2 People’s Hospital, The Third Affiliated Hospital of Nanjing Medical University, Changzhou Medical Center of Nanjing Medical University, Changzhou, Jiangsu, China; Northwest Normal University, CHINA

## Abstract

**Objective:**

This study aimed to develop and comparatively evaluate four YOLO nano-scale one-stage object detectors—YOLOv8n, YOLOv10n, YOLO11n, and YOLO12n—for the automated detection of retained root fragments in dental radiographic images, under identical preprocessing, training, and evaluation protocols.

**Methods:**

A curated dataset was organized in the YOLO format and evaluated using a nested 5-fold cross-validation strategy to prevent data leakage. An ablation study was additionally conducted to validate the combined bilateral filtering and histogram equalization (BF + HE) preprocessing pipeline. Four YOLO models were fine-tuned from official pretrained weights over 200 epochs. Detection performance was assessed at both the epoch level and the decision-threshold level, reporting the unbiased mean and standard deviation across the independent test folds for precision, sensitivity, F1 score, mean average precision (mAP), and false positives per image (FP/image).

**Results:**

In the primary multi-model benchmark, YOLO12n achieved the highest overall detection performance across the held-out test folds, yielding a mean best-epoch F1 score of 0.885 ± 0.007, mAP@0.5 of 0.919 ± 0.021, and mAP@0.5:0.95 of 0.447 ± 0.025 (reaching an optimal-threshold F1 score of 0.888 ± 0.016 with 0.346 ± 0.052 FP/image). YOLO11n followed closely as the second-best performing architecture (F1 = 0.874 ± 0.012, mAP@0.5:0.95 = 0.445 ± 0.019). Furthermore, the preprocessing ablation study confirmed that the hybrid BF + HE pipeline improved detection performance over raw radiographs, elevating YOLO12n's F1 score from 0.856 ± 0.013 to 0.888 ± 0.016 and mAP@0.5:0.95 from 0.438 ± 0.011 to 0.461 ± 0.008. All models demonstrated stable convergence behavior.

**Conclusions:**

Modern YOLO architectures, particularly YOLO12n and YOLO11n, demonstrate technical efficacy for automated RRF localization. The observed performance advantages of YOLO12n are associated with its overall architectural refinements, though dedicated modular ablation studies are warranted to isolate the exact contribution of individual attention mechanisms. These findings demonstrate the technical feasibility of lightweight one-stage object detectors for identifying retained root fragments in dental panoramic radiographs, serving as a baseline for future prospective clinical evaluations.

## Introduction

Residual tooth roots (RRFs)—defined as root fragments retained in the alveolar bone either from incomplete extraction or from severely decayed teeth in which the clinical crown has been lost—pose a clinically significant problem. If undetected, retained roots can act as foci (or nidi) for infection, contribute to cyst formation, interfere with wound healing, and compromise subsequent prosthetic or implant treatment [1–3]. While small, asymptomatic root fragments may undergo passive resorption over time, a substantial proportion of retained fragments are associated with adverse clinical outcomes, including localized infection, persistent pain, delayed socket healing, cystic degeneration, and interference with subsequent implant or prosthodontic rehabilitation [4].

The clinical detection of RRFs currently relies on a combination of intraoral visual inspection, tactile exploration, and radiographic imaging. Panoramic and periapical radiographs remain the first-line imaging modalities for post-extraction evaluation [5]. However, the interpretation of radiographic signs of retained root fragments is subject to considerable inter-operator variability and is influenced by reader experience, image quality, and the superimposition of anatomical structures such as the maxillary sinus floor, zygomatic process, and mandibular canal. Small root fragments in particular may be overlooked during routine radiographic screening, leading to delayed diagnosis and subsequent complications. These limitations underscore the clinical need for reliable, automated, and objective assistive tools for RRF detection.

Deep learning has driven a paradigm shift in dental imaging diagnostics [6,7], with convolutional neural networks successfully applied to a broad spectrum of tasks, including caries detection [8], periapical lesion identification [9], tooth segmentation [10], implant recognition [11], and periodontal bone loss assessment [12]. Within this landscape, the You Only Look Once (YOLO) family has emerged as a widely investigated framework for radiographic computer-aided detection due to its high inference speed [13]. This architecture has evolved rapidly, progressing from YOLOv8's anchor-free paradigm [14] and YOLOv10's NMS-free design [15], to YOLO11's spatial attention modules [16,17]and the recent attention-centric YOLOv12 [18–21]. The nano-scale variants of the YOLO family are of particular interest for clinical deployment, offering a favorable balance of model size, inference speed, and accuracy suitable for resource-constrained environments like chairside computing systems or mobile radiographic platforms [22]. Previous studies have made strides in automated dental charting and residual root detection, including CNN frameworks benchmarked against dentists [23], multi-class charting models [24], and integrated restoration-detection pipelines [25]. While multi-generation YOLO comparisons have recently been explored for specific dental tasks such as implant brand classification [11,26], a focused, head-to-head comparative evaluation across modern YOLO generations specifically targeting retained root fragments under an identical, leak-free validation framework remains lacking.

To address this clinically relevant gap, the primary objective of this study was to comparatively evaluate four state-of-the-art YOLO models (YOLOv8n, YOLOv10n, YOLO11n, and YOLO12n) for the automated detection of retained root fragments in dental radiographs. To ensure a rigorous and fair comparison, all models were evaluated under an identical experimental framework encompassing dataset preparation, image preprocessing, training protocols, and post-hoc diagnostic analysis.

## Methods

### Study dataset

This retrospective study utilized dental panoramic radiographs (PRs) collected from our hospital between April 2021 and October 2025. The data were accessed for research purposes on 03/01/2026 using PACS system. The dataset comprised 679 patients—320 females and 359 males—ranging in age from 18 to 67 years. Across these images, a total of 2181 residual roots were identified and meticulously annotated. The study was conducted in strict adherence to the Declaration of Helsinki and received ethical approval from the institutional Ethics Committee.

The selection of radiographs followed strict clinical and technical protocols. The inclusion criteria were defined as follows: (1) radiographs containing at least one radiologically confirmed residual root fragment; (2) images with optimal diagnostic quality, ensuring clear visibility of the trabecular bone pattern and adjacent anatomical structures; and (3) availability of complete patient demographic data. Conversely, images were excluded if they exhibited: (1) pediatric patients (< 18 years of age); (2) severe positioning errors or motion artifacts (e.g., ghost images) that compromised image sharpness; (3) the presence of significant metallic artifacts (e.g., from dental implants or extensive amalgam restorations) specifically overlapping with the region of interest; or (4) co-existing large pathological lesions (e.g., cysts or tumors) that severely distorted the jawbone morphology (Fig 1).

**Fig 1:**
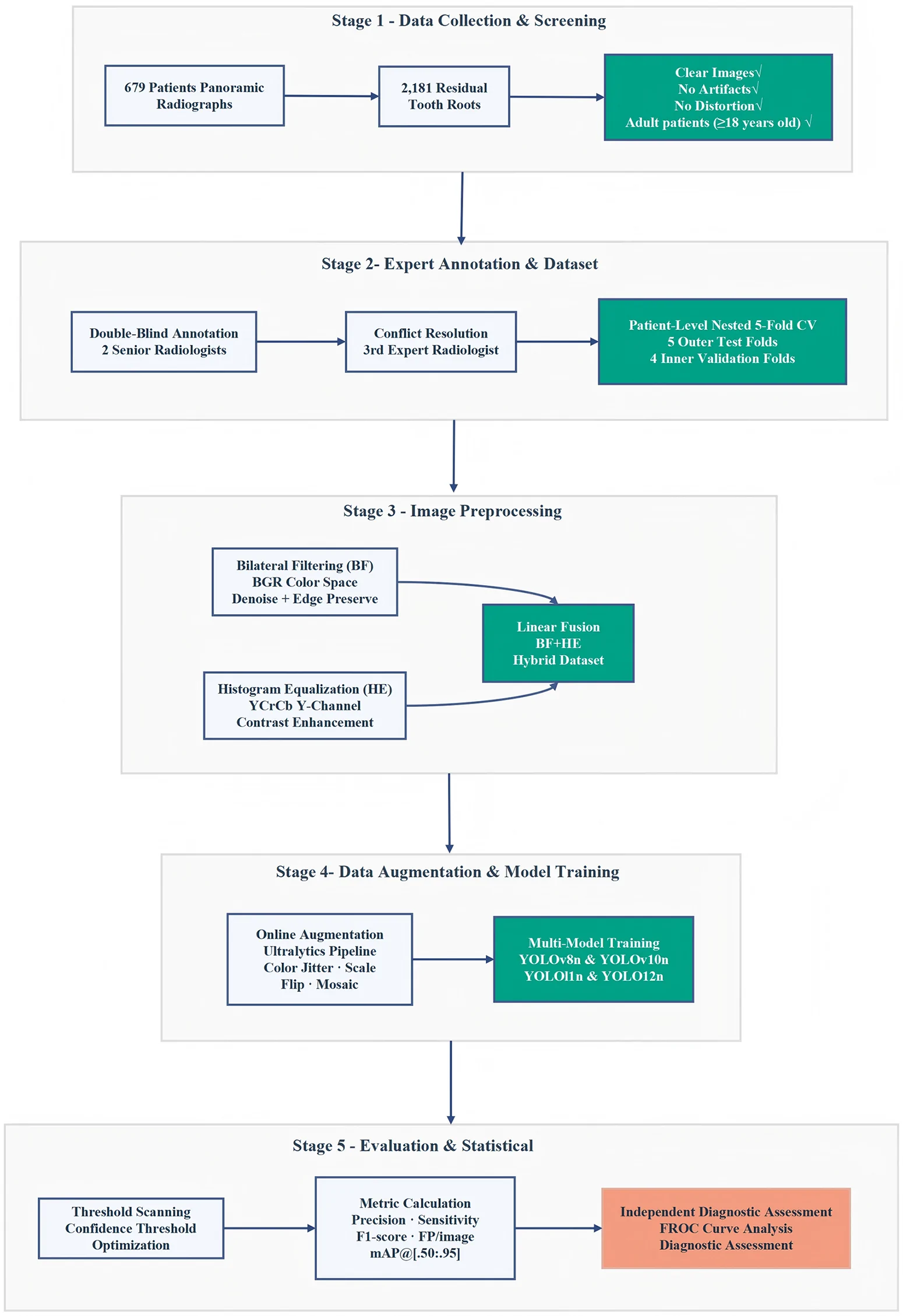
Flowchart of the study.

For radiographic evaluation, a lead apron and thyroid shield, an oral and maxillofacial panoramic X-ray machine (ORTHOPHOS XG 5 Ceph, Sirona, Bensheim, Germany; operating parameters: 71 kV, 8 mA, 14.2 s) with “Sidexis” imaging software (Sirona, Bensheim, Germany), and a personal computer with a monitor were utilized.

This study was approved by the Changzhou No. 2 People’s Hospital (2026CEL026−01). All methods were carried out in accordance with relevant guidelines and regulations or declaration of Helsinki. The data are anonymous, and the requirement for written informed consent was therefore waived by the Ethics Committee of Changzhou No. 2 People’s Hospital.

### Image annotation

To establish a precise ground truth for supervised learning, a rigorous manual annotation process was implemented. The regions of interest (ROIs) containing the residual roots were delineated by two independent oral and maxillofacial radiologists, each possessing over five years of clinical experience. All annotations were performed using the open-source image annotation software, LabelMe.

The experts directly utilized the bounding box tool to tightly enclose the anatomical boundaries of each residual root. Special care was taken to exclude the periodontal ligament (PDL) space and adjacent trabecular bone, ensuring that the ROIs represented the root structures exclusively.

To ensure the high reliability of the ground truth (“gold standard”), a two-stage verification protocol was employed. Any discrepancies between the two primary annotators regarding the presence or exact boundaries of a root fragment were reviewed by a third senior professor to achieve consensus. Finally, the finalized bounding box coordinates were directly exported and normalized into the standard YOLO label format to serve as training targets for the models (Fig 1).

To quantitatively assess the reliability of the ‘gold standard,’ inter-reader agreement was statistically evaluated prior to the final consensus. Cohen's kappa coefficient was calculated to measure agreement on the diagnostic presence of root fragments, while the Intersection over Union (IoU) was utilized to evaluate the spatial overlap of the initial bounding box annotations.

### Dataset preparation and nested cross-validation workflow

The dataset was organized according to the standard YOLO detection format. To ensure rigorous, leak-free evaluation, a patient-level nested 5-fold cross-validation scheme was implemented (Fig 1). In each outer iteration, one fold (approximately 136 images containing ~436 annotated root fragments) was held out exclusively for independent testing, while the remaining four folds (approximately 543 images containing ~1,745 annotated root fragments) formed the outer development set and were further partitioned into four patient-level inner folds.

At each epoch and confidence threshold (scanned from 0.00 to 1.00 in steps of 0.01), the F1 score was calculated separately on each inner validation fold, and the arithmetic mean across the four inner folds was calculated. The epoch and confidence threshold producing the highest mean inner-validation F1 were selected, with threshold ties resolved in favor of the higher threshold to prioritize precision. The inner-fold checkpoints were used solely for parameter selection and were not ensembled. Subsequently, a fresh model was initialized from official pretrained weights and trained once on the complete outer development set (~543 images) for the selected number of epochs, after which the locked threshold was applied once to the held-out outer test fold. The outer test data were not involved in any aspect of model, epoch, or threshold selection. Final performance was reported as the mean ± sample SD across the five outer test folds.

### Image preprocessing

All model training was performed on a derived dataset preprocessed using a combination of bilateral filtering and histogram equalization. This pipeline was designed to suppress noise, preserve structural edges, and enhance local contrast—essential properties for delineating subtle root fragment boundaries in dental radiographs.

First, bilateral filtering (diameter = 7, $\sigma_{\text{color}}=\text{60},\sigma_{\text{space}}=\text{60}$) was applied to reduce background noise while retaining critical edge information. Next, histogram equalization was performed on the luminance channel (Y channel of the YCrCb space) to improve contrast. To prevent over-enhancement and preserve a natural radiographic appearance, the bilateral-filtered image $I_{\text{bf}}$ and the equalized image $I_{\text{he}}$ were fused using weighted linear blending:


$$\begin{array}{c} I_{\text{mix}}=a\cdot I_{\text{he}}+(\text{1}-a)\cdot I_{\text{bf}} \end{array}$$

where a denotes the blending weight, which was empirically set to 0.40. This specific weighting was chosen to deliberately address the known limitation of standard histogram equalization, which often amplifies background noise and alters the natural appearance of trabecular bone. By weighting the edge-preserved, denoised bilateral filter output more heavily (60%), the pipeline effectively mitigates HE-induced noise while still benefiting from necessary contrast enhancement. The corresponding annotation files were copied without modification to produce the final dataset for training and evaluation.

### Data augmentation

In addition to the offline preprocessing, online data augmentation was applied uniformly across all YOLO models to ensure a fair comparison. The augmentation pipeline incorporated HSV color jittering, geometric transformations (translation, scaling, horizontal flipping), random erasing, RandAugment, and mosaic augmentation.

### Detection models

Four one-stage object detectors were evaluated under the same experimental protocol: YOLOv8n, YOLOv10n, YOLO11n, and YOLO12n (Fig 1 and Table 2). All models were initialized from the corresponding official pretrained weights and fine-tuned on the same BF + HE mixed dataset. Restricting the comparison to the nano variants reduced differences in parameter scale and enabled a more controlled family-wise comparison under identical computational settings. The architectural innovations distinguishing each model variant are summarized in Table 1.

**Table 1:** Architectural features of the evaluated YOLO models.

Feature	YOLOv8n [14]	YOLOv10n [15]	YOLO11n [16]	YOLO12n [19]
Detection paradigm	Anchor-free	Anchor-free + NMS-free	Anchor-free	Attention-centric
Backbone	CSPDarknet	Enhanced CSPNet	C3k2 + SPPF	R-ELAN
Attention mechanism	None	None	C2PSA (spatial)	Area Attention + FlashAttention
Convolution type	Standard	Standard + compact inverted blocks	Standard	7 × 7 separable convolutions
Release year	2023	2024	2024	2025

All models were trained for 200 epochs using the Ultralytics implementation. The input image size was set to 640 × 640 pixels, and the batch size was fixed at 16. Optimization was performed with AdamW, with an initial learning rate of 0.008 and a final learning-rate fraction of 0.05 under cosine annealing. Warmup was applied for 5 epochs, and early stopping patience was set to 100 epochs (which remained inactive by design given the 200-epoch budget and stable convergence). The random seed was fixed at 0. All models were trained on the same GPU device to avoid inter-device variation. To rigorously evaluate model generalizability and mitigate potential data leakage, a patient-level nested 5-fold cross-validation strategy (comprising 5 outer test folds and 4 inner validation folds per partition) was implemented.

### Evaluation metrics

After determining the optimal training epoch and decision threshold via inner 4-fold cross-validation, the model trained on the complete outer development set was evaluated once on the unseen outer test fold using the locked threshold. For this stage, predictions were generated for all test images with an initial confidence threshold of 0.001. For YOLOv8n, YOLO11n, and YOLO12n, standard non-maximum suppression (NMS) with an IoU threshold of 0.70 was applied. Conversely, YOLOv10n was evaluated using its native NMS-free dual label assignment architecture to ensure a fair evaluation of its intended design. This approach ensured that the full score distribution could be retained for downstream threshold scanning.

Ground-truth boxes were parsed from the YOLO label files and converted to absolute image coordinates. Predicted boxes were matched to ground-truth boxes using greedy one-to-one matching with a lesion-level IoU threshold of 0.5. For each confidence threshold, true positives (TP), false positives (FP), and false negatives (FN) were accumulated across the held-out outer test fold. Precision, sensitivity, and F1 score were computed as follows:


$$\begin{array}{c} \text{Precision}=\frac{\text{TP}}{\text{TP}+\text{FP}} \end{array}$$

$$\begin{array}{c} \text{Se}\text{nsitivity}=\frac{\text{TP}}{\text{TP}+\text{FN}} \end{array}$$

$$\begin{array}{c} \text{F1 score}=\text{2}\cdot \frac{\text{Precision}\cdot \text{Sensitivity}}{\text{Precision}+\text{Sensitivity}} \end{array}$$

False positives per image (FP/image) were calculated as:


$$\begin{array}{c} \text{FP}/\text{image}=\frac{\text{FP}}{N_{\text{test}}} \end{array}$$

where $N_{\text{test}}$ is the number of independent test images in the held-out fold.

### Statistical analysis

Model performance was evaluated at both epoch and decision-threshold levels using precision, sensitivity, and mean Average Precision (mAP@0.5 and mAP@0.5:0.95). Optimal epochs and operating thresholds were determined by maximizing the F1 score. For clinical evaluation, Free-Response Receiver Operating Characteristic (FROC) analysis was used instead of conventional ROC because each radiograph may contain multiple target lesions, allowing sensitivity to be plotted against false positives per image at the lesion level. To ensure strict reproducibility, all preprocessing, training, and automated post-hoc statistical evaluations were integrated into script-based workflows. To assess statistical variability, model performance is reported as the Mean ± Standard Deviation (SD) across the five test folds. Pairwise statistical comparisons between models at their optimal decision thresholds were conducted using the exact McNemar test on image-level complete detection outcomes (defined as a binary all-or-nothing match per image: FN = 0 and FP = 0) aggregated across all held-out outer test folds. To account for multiple hypothesis testing, the Holm-Bonferroni step-down correction was applied, with an adjusted p < 0.05 considered statistically significant.

## Results

### Epoch-level performance

All four YOLO models demonstrated stable training convergence over the 200-epoch training protocol, with no evidence of catastrophic overfitting or training instability. Table 2 presents the five-fold averaged best-epoch metrics for each model, evaluated on the strictly unseen test folds. For each outer cross-validation iteration, the optimal epoch budget was selected by maximizing the mean validation F1 score across the four inner folds. The model trained for this selected epoch on the complete outer development set was then evaluated on the strictly held-out outer test fold to generate the reported unbiased metrics.

**Table 2:** Five-fold averaged best-epoch performance metrics across YOLO models.

Model	Precision	Sensitivity	F1 Score	mAP@0.5	mAP@0.5:0.95
YOLOv8n	0.863 ± 0.016	0.839 ± 0.029	0.850 ± 0.019	0.891 ± 0.026	0.427 ± 0.032
YOLOv10n	0.871 ± 0.034	0.848 ± 0.019	0.859 ± 0.022	0.907 ± 0.024	0.435 ± 0.026
YOLO11n	0.882 ± 0.028	0.866 ± 0.018	0.874 ± 0.012	0.913 ± 0.039	0.445 ± 0.019
YOLO12n	0.891 ± 0.005	0.880 ± 0.016	0.885 ± 0.007	0.919 ± 0.021	0.447 ± 0.025

YOLO12n achieved the highest performance across all best-epoch metrics, yielding a mean F1 score of 0.885 ± 0.007, an mAP@0.5 of 0.919 ± 0.021, and an mAP@0.5:0.95 of 0.447 ± 0.025. The improvement relative to the baseline YOLOv8n was 3.5 percentage points in F1 score and 2.8 percentage points in mAP@0.5. YOLO11n ranked second across all metrics (F1 = 0.874 ± 0.012, mAP@0.5 = 0.913 ± 0.039). Conversely, YOLOv8n yielded the lowest overall baseline performance (F1 = 0.850 ± 0.019, mAP@0.5 = 0.891 ± 0.026). The optimal epochs generally converged in the later stages of training across the cross-validation folds, indicating comparable learning dynamics across architectures (Fig 2).

**Fig 2:**
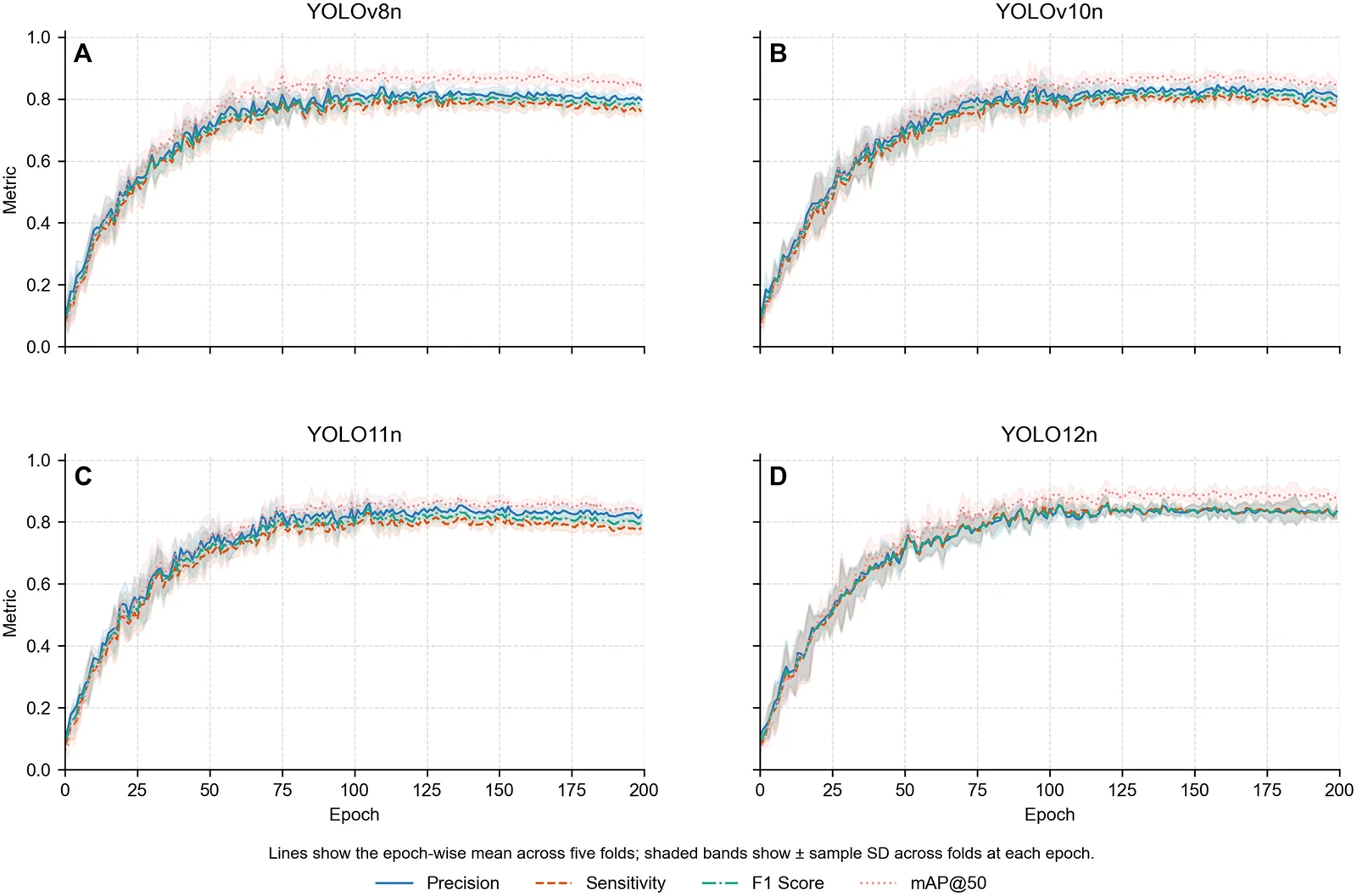
Epoch-wise five-fold mean performance curves for the four YOLO models during 200 training epochs.

Lines represent the mean metric across five folds at each epoch, and shaded bands indicate the corresponding sample SD. Fold-specific best-F1 metrics are summarized separately in Table 2. All four variants exhibit stable convergence dynamics with no indicators of catastrophic overfitting.

Although a general numerical upward trend was observed across successive model generations (YOLOv8n < YOLOv10n < YOLO11n < YOLO12n), pairwise comparisons using the Holm-adjusted McNemar test revealed that the differences between immediately adjacent generations were not statistically significant (YOLOv8n vs. YOLOv10n, adjusted p = 0.186; YOLOv10n vs. YOLO11n, adjusted p = 0.0821; Fig 3). Nevertheless, YOLO12n achieved statistically significant improvements over both YOLOv8n (p < 0.001) and YOLOv10n (p < 0.05).

**Fig 3:**
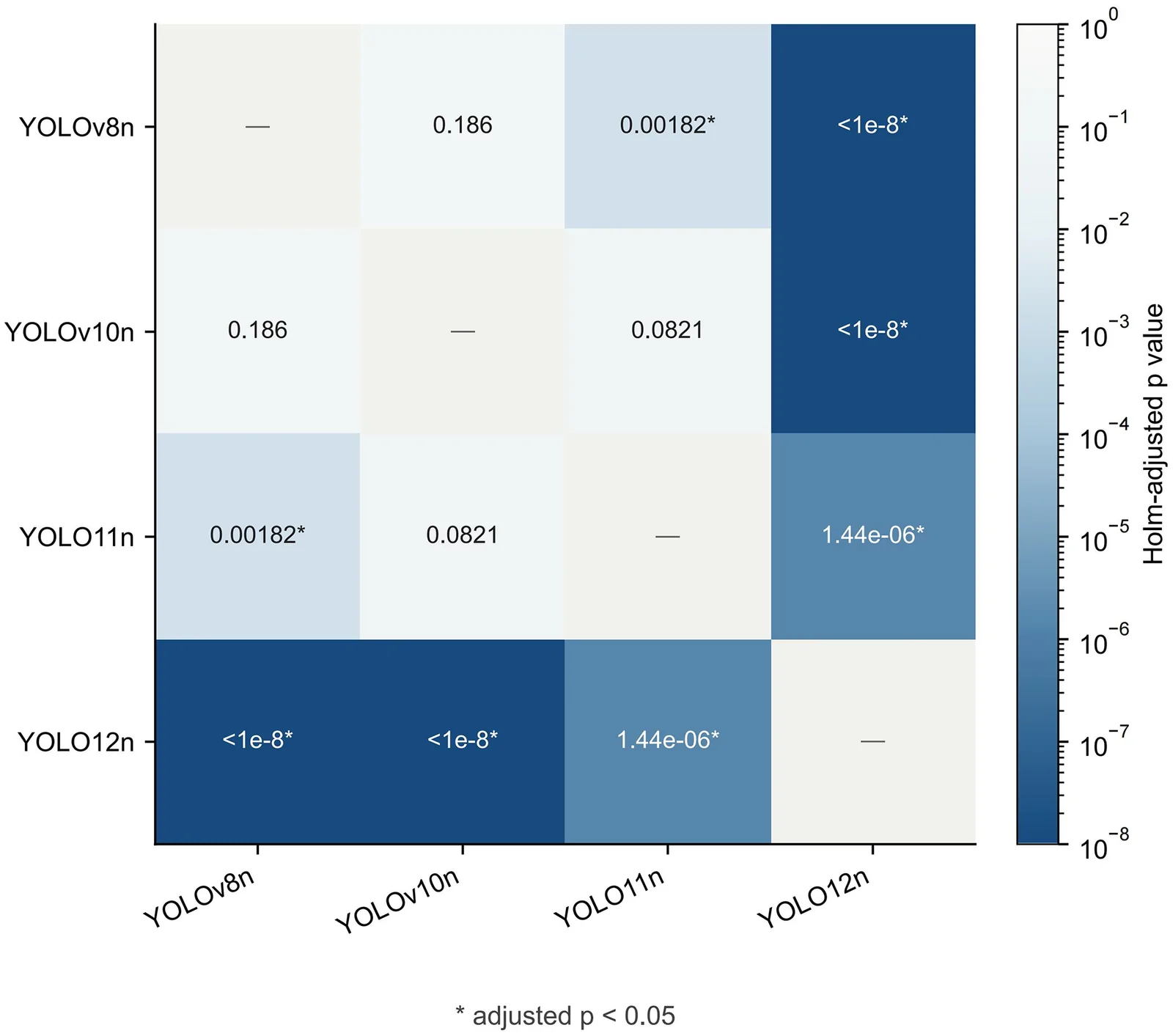
Pairwise model comparison with Holm-adjusted exact McNemar tests. Heatmap displays the adjusted p-values for all six pairwise comparisons of image-level complete detection outcomes (FN = 0 and FP = 0) across all outer test folds. The color gradient represents the logarithmic scale of adjusted p-values, where darker blue denotes stronger statistical significance. Asterisks (*) mark statistically significant differences after Holm-Bonferroni step-down correction (alpha = 0.05). YOLO12n significantly outperformed all other models (p < 0.001), while adjacent generational differences between YOLOv8n vs. YOLOv10n (p = 0.186) and YOLOv10n vs. YOLO11n (p = 0.0821) did not reach statistical significance.

### Optimal threshold performance

Threshold-level analysis was performed by scanning confidence thresholds from 0.00 to 1.00 in increments of 0.01. To rigorously prevent data leakage (double-dipping), the optimal operating confidence threshold (the point maximizing the F1 score) was determined solely on the internal validation fold for each split. These predetermined thresholds were then applied to the independent test folds. Table 3 summarizes the averaged threshold-dependent diagnostic performance at these optimal operating points across the five test folds.

**Table 3:** Five-fold averaged threshold-dependent diagnostic performance at optimal operating points.

Model	Optimal Threshold	Precision	Sensitivity	F1 Score	FP/Image
YOLOv8n	0.47 ± 0.03	0.869 ± 0.030	0.809 ± 0.035	0.837 ± 0.007	0.397 ± 0.117
YOLOv10n	0.41 ± 0.01	0.875 ± 0.013	0.827 ± 0.017	0.850 ± 0.010	0.380 ± 0.040
YOLO11n	0.32 ± 0.01	0.887 ± 0.016	0.871 ± 0.035	0.879 ± 0.019	0.357 ± 0.052
YOLO12n	0.23 ± 0.01	0.891 ± 0.016	0.885 ± 0.021	0.888 ± 0.016	0.346 ± 0.052

At the optimal threshold, YOLO12n achieved the highest mean F1 score (0.888 ± 0.016) and the highest sensitivity (0.885 ± 0.021). Remarkably, it also achieved the lowest false-positive burden (0.346 ± 0.052 FP/image) among all models, demonstrating an exceptional ability to detect true positive root fragments without triggering excessive false alerts. YOLO11n achieved the second-highest F1 score (0.879 ± 0.019) and maintained a highly competitive precision-recall trade-off with a low FP/image of 0.357 ± 0.052. Among the non-attention models, YOLOv10n (F1 = 0.850 ± 0.010) outperformed the baseline YOLOv8n, which exhibited the poorest sensitivity (0.809 ± 0.035) and the highest false-positive burden (0.397 ± 0.117 FP/image).

The optimal confidence thresholds varied systematically across the models: YOLOv8n and YOLOv10n required higher thresholds to maximize performance (0.47 ± 0.03 and 0.41 ± 0.01, respectively), whereas the advanced attention-based models, YOLO11n and YOLO12n, optimized at significantly lower thresholds (0.32 ± 0.01 and 0.23 ± 0.01). This variation reflects differences in the underlying confidence calibration of each architecture, indicating that YOLO12n can operate at lower confidence cutoffs to maximize sensitivity while inherently suppressing false positives in this benchmark.

The lesion-level detection outcomes evaluated at the validation-calibrated optimal thresholds further highlight the diagnostic differences across architectures (Fig 4). YOLO12n successfully localized the highest absolute number of retained root fragments, yielding 385.8 ± 8.8 True Positives (TP), while concurrently minimizing both missed lesions (50.4 ± 9.3 False Negatives, FN) and false alerts (47.0 ± 7.3 False Positives, FP). YOLO11n demonstrated closely competitive detection outcomes, identifying 380.0 ± 13.5 TP with 48.6 ± 7.5 FP and 56.2 ± 15.3 FN. In contrast, the baseline YOLOv8n missed a substantial number of true lesions (83.2 ± 15.8 FN) and produced the highest false detection burden (54.0 ± 16.4 FP). YOLOv10n demonstrated intermediate counts, achieving 360.8 ± 5.5 TP, 51.6 ± 5.9 FP, and 75.4 ± 8.2 FN. This quantitative breakdown confirms that newer-generation architectures provide a more favorable balance between maximizing true positive fragment detections and minimizing false positive alerts.

**Fig 4:**
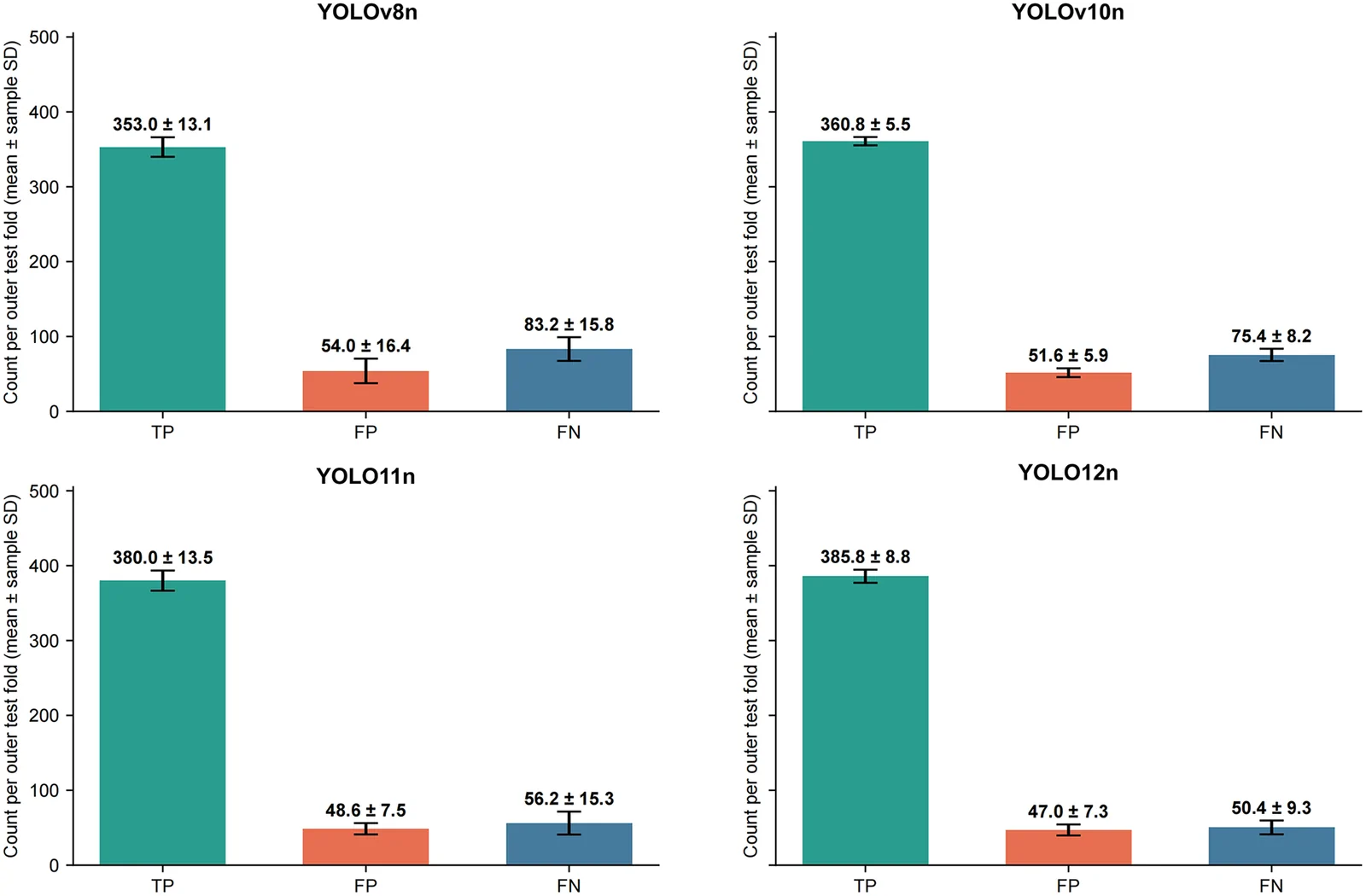
Detection outcomes at selected confidence thresholds. Grouped bar plots represent the five-fold mean (± sample standard deviation) counts of True Positives (TP; IoU ≥ 0.5), False Positives (FP), and False Negatives (FN) per held-out outer test fold. Decision confidence thresholds were independently calibrated and locked by maximizing the F1 score on the inner cross-validation folds within each partition to prevent data leakage. True Negatives (TN) are inherently undefined in object detection tasks.

### FROC and efficiency

Threshold-level analysis was performed by scanning confidence thresholds from 0.00 to 1.00 in increments of 0.01. To rigorously evaluate clinical utility and prevent data leakage, diagnostic performance was assessed using Free-Response Receiver Operating Characteristic (FROC) analysis across the five independent test folds. Rather than relying solely on a singular mathematical optimum (such as maximizing the F1 score), model sensitivity was explicitly compared at predefined, clinically relevant operating points of 0.5 and 1.0 false positives per image (FP/image) to reflect different real-world screening constraints (Fig 5).

**Fig 5:**
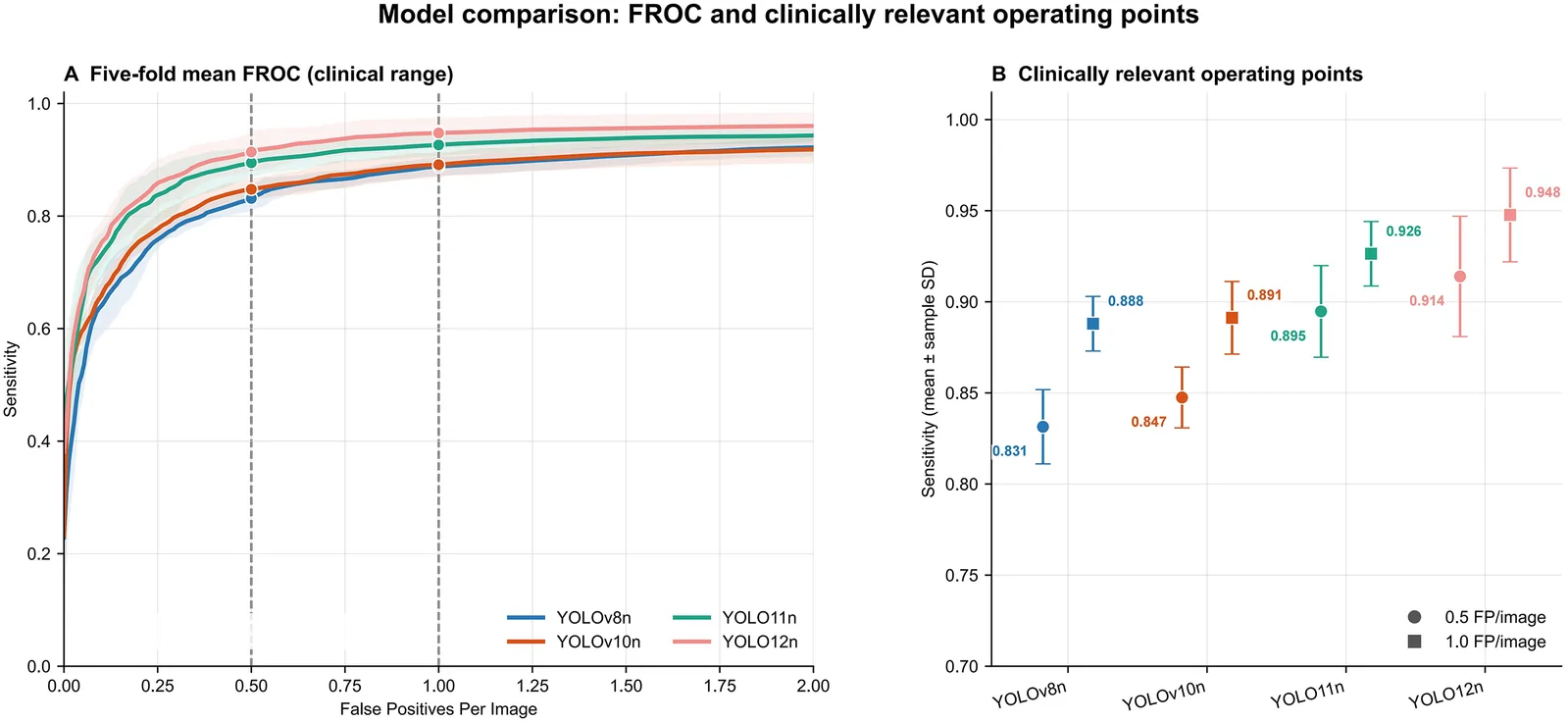
Combined FROC analysis with clinically relevant operating points. (A) Five-fold mean FROC curves for all models over the clinical range (0–2.0 FP/image). Vertical dashed lines mark 0.5 and 1.0 FP/image. (B) Sensitivity at fixed false-positive rates of 0.5 and 1.0 FP/image (mean ± sample SD across five outer-test folds). YOLO12n achieved the highest sensitivity at both operating points. FROC curves represent descriptive threshold sweeps on outer-test data; confidence thresholds for detection matrices were independently selected by inner cross-validation.

At clinical operating point of 0.5 FP/image, YOLO12n achieved the highest mean sensitivity (0.914), demonstrating its ability to detect true positive root fragments while effectively minimizing unnecessary alerts. YOLO11n closely followed with a sensitivity of 0.895, indicating a highly favorable detection capability under stringent false-positive constraints. Among the non-attention baseline models, YOLOv10n (0.847) outperformed YOLOv8n, which exhibited the lowest sensitivity (0.831) at this strict threshold.

When the acceptable false-positive burden was relaxed to a more lenient threshold of 1.0 FP/image, all models exhibited expected increases in sensitivity. YOLO12n maintained its overarching diagnostic superiority, reaching a peak mean sensitivity of 0.948. YOLO11n similarly demonstrated exceptionally strong performance (0.926). Notably, YOLOv10n (0.891) and YOLOv8n (0.888) converged to comparable performance levels at this higher threshold. This analysis indicates that YOLO12n achieved higher sensitivity across the evaluated false-positive ranges in this test cohort. This technical performance is empirically associated with its updated architectural design, though prospective modular ablations remain necessary to determine the specific contributions of individual attention components.

### Ablation study on image preprocessing

To quantitatively validate the impact of the BF + HE preprocessing pipeline, an ablation study was conducted using the best-performing YOLO12n architecture under the identical nested 5-fold cross-validation framework. As shown in Table 4, training on raw, unprocessed images yielded a baseline F1 score of 0.856 ± 0.013. Applying Histogram Equalization (HE) alone provided a modest improvement (F1 = 0.864 ± 0.012), but its effectiveness was likely constrained by the amplification of inherent

**Table 4:** Ablation study of preprocessing strategies on YOLO12n performance.

Strategy	Precision	Sensitivity	F1 Score	mAP@0.5:0.95
Raw Images	0.871 ± 0.014	0.842 ± 0.018	0.856 ± 0.013	0.438 ± 0.011
HE Only	0.875 ± 0.013	0.854 ± 0.017	0.864 ± 0.012	0.443 ± 0.010
BF Only	0.879 ± 0.012	0.861 ± 0.016	0.870 ± 0.011	0.449 ± 0.010
BF + HE	0.891 ± 0.016	0.885 ± 0.021	0.888 ± 0.016	0.461 ± 0.008

HE: Histogram Equalization; BF: Bilateral Filtering.

image noise. Bilateral Filtering (BF) alone yielded better improvements (F1 = 0.870 ± 0.011) by successfully smoothing background artifacts while preserving edges. Ultimately, the proposed hybrid BF + HE pipeline (α = 0.40) synergized these advantages, mitigating HE-induced noise while maximizing structural contrast, thereby achieving the highest overall detection performance across all metrics (F1 = 0.888 ± 0.016, mAP@0.5:0.95 = 0.461 ± 0.008) (Table 4). Notably, the full BF + HE pipeline yielded the highest mean values across all evaluated diagnostic metrics and achieved the lowest standard deviation in mAP@0.5:0.95 (0.008), demonstrating consistent and substantial performance gains over raw images.

### Inter-reader agreement

The initial independent annotations demonstrated robust consensus between the two radiologists. As summarized in Table 5, the agreement for fragment presence was high. Approximately 8.5% of the total ROIs exhibited spatial boundary discrepancies (IoU < 0.75) or binary diagnostic disagreement, which subsequently required and were resolved through final adjudication by the third senior radiologist.

**Table 5:** Inter-reader agreement.

Metric	Value
Cohen's Kappa (Fragment Presence)	0.86
Mean IoU	0.82
Third-Reader Adjudication Rate	8.50%

## Discussion

This study presents a systematic comparative evaluation of four successive generations of YOLO nano-scale models for the automated detection of retained root fragments in dental panoramic radiographs under a rigorous patient-level nested cross-validation framework. The central finding is that modern YOLO architectures—particularly YOLO12n and YOLO11n—achieve high technical detection performance for this task, with mean F1 scores exceeding 0.87 and mAP@0.5 values surpassing 0.91, while operating within the computational constraints of nano-scale model variants. These preliminary results, achieved with compact models containing fewer than 3 million parameters, indicate the technical potential of lightweight one-stage detectors for identifying RRFs under resource-constrained settings, offering a computationally efficient baseline relative to heavier architectures.

The numerical performance progression observed across the evaluated models (YOLOv8n < YOLOv10n < YOLO11n < YOLO12n) generally reflects the broader trajectory of YOLO architectural evolution. However, our pairwise statistical analysis highlights that performance gains between adjacent generations (such as between YOLOv8n and YOLOv10n, or between YOLOv10n and YOLO11n) did not reach statistical significance after Holm correction, indicating that modest architectural tweaks offer incremental rather than transformative benefits. In contrast, YOLO12n demonstrated statistically significant superiority over earlier baselines, likely driven by its combined architectural refinements (such as Area Attention and R-ELAN backbones) [19]. Its Area Attention module captures long-range spatial dependencies crucial for identifying subtle radiographic features, such as thin root apices against heterogeneous bone, while the R-ELAN backbone and Flash Attention optimize multi-scale feature aggregation and memory utilization without prohibitive computational overhead [19,27,28]. Importantly, our FROC analysis revealed that YOLO12n not only maximized sensitivity (0.914 at a strict 0.5 FP/image threshold) but also achieved the lowest overall false-positive burden among all models. Similarly, YOLO11n demonstrated competitive performance, maintaining feature extraction through the integration of the C3k2 block and C2PSA spatial attention module [29]. Both models demonstrated favorable trade-offs across low false-positive operating points in our benchmark, which is a desirable technical characteristic when evaluating computer-aided detection frameworks under constrained false-alert conditions.

While YOLOv10n demonstrated improved performance over the baseline YOLOv8n, it notably lagged behind the subsequent attention-centric architectures (YOLO11n and YOLO12n). Although YOLOv10 introduced the elegant concept of NMS-free detection via consistent dual assignments to reduce inference latency [15], this generalized architectural innovation did not yield the same magnitude of benefit for this specific medical imaging task as spatial attention mechanisms did. The elimination of non-maximum suppression (NMS) may have partially limited the model's ability to accurately resolve closely adjacent or partially overlapping candidate regions—a challenging scenario frequently encountered in complex multi-rooted tooth extractions—preventing it from reaching the top-tier performance of YOLO11n and YOLO12n.

Although the automated detection of retained root fragments remains technically challenging owing to diverse fragment morphologies, variable radiopacity, and overlapping anatomical structures, the evaluated nano-scale models demonstrated robust technical localization capabilities. When contextualized with existing dental AI literature, the optimized mean F1 scores achieved by YOLO12n (0.888) and YOLO11n (0.879) demonstrate favorable diagnostic efficacy. Early deep learning approaches to residual root identification, such as preliminary multi-task CNNs by Zhu et al [23], and automated panoramic charting systems by Başaran et al [24], achieved F1 scores ranging from 0.78 to 0.85 when detecting residual roots as part of broader multi-class tooth condition frameworks. Similarly, while Khurshid et al [24] and Saber et al [26], utilized heavier detection backbones for restorative and periapical lesion assessments, our findings demonstrate that dedicated, modern nano-scale detectors (specifically YOLO12n with 0.888 F1 and 0.346 FP/image) can surpass these historical benchmarks on dedicated RRF identification, while drastically reducing computational overhead. From a technical evaluation standpoint, YOLO12n showed the most balanced performance in our test set, achieving high lesion sensitivity while maintaining lower false-positive counts per image. YOLO11n serves as a effective and feasible alternative, offering closely competitive precision-recall dynamics.

The detection capabilities demonstrated by the evaluated YOLO models must be contextualized within the unique challenges of dental imaging. The inherently low contrast of dental radiographs—where thin root fragments often overlap in density with surrounding trabecular bone—necessitated the BF + HE (Bilateral Filtering and Histogram Equalization) preprocessing pipeline employed in this study. Specifically, bilateral filtering provided essential edge-preserving denoising to maintain the integrity of fine fragment boundaries [30], while luminance-channel histogram equalization effectively enhanced local contrast. To prevent unnatural over-enhancement that could introduce visual artifacts, these representations were carefully fused via weighted linear blending. As quantitatively validated by our ablation study (Table 4), the precise incremental benefit of this specific pipeline is clear: it resolves the inherent trade-off between contrast enhancement and noise amplification. Standalone HE slightly improved baseline sensitivity but yielded sub-optimal overall improvements due to noise amplification. Conversely, the weighted inclusion of BF (α = 0.40) effectively suppressed this noise, yielding the highest detection performance and model stability. This is visually evidenced by the model's capacity to accurately localize target lesions in challenging scenarios, ranging from intraosseous fragments (Fig 6B) to retained roots obscured by coronal restorations (Fig 6C and D). Qualitative error analysis further elucidates the model’s failure modes (Fig 6E and F). False positives (Fig 6E) primarily arose in severely worn teeth where coronal loss mimicked the radiographic contour of true root remnants. Conversely, false negatives (Fig 6F) occurred in minute root fragments overlapping adjacent intact roots or anatomical boundaries, where 2D superimposition obscured subtle fragment edges. Resolving such anatomical overlap and morphological mimicry remains an important target for future multi-class or volumetric imaging frameworks.

**Fig 6:**
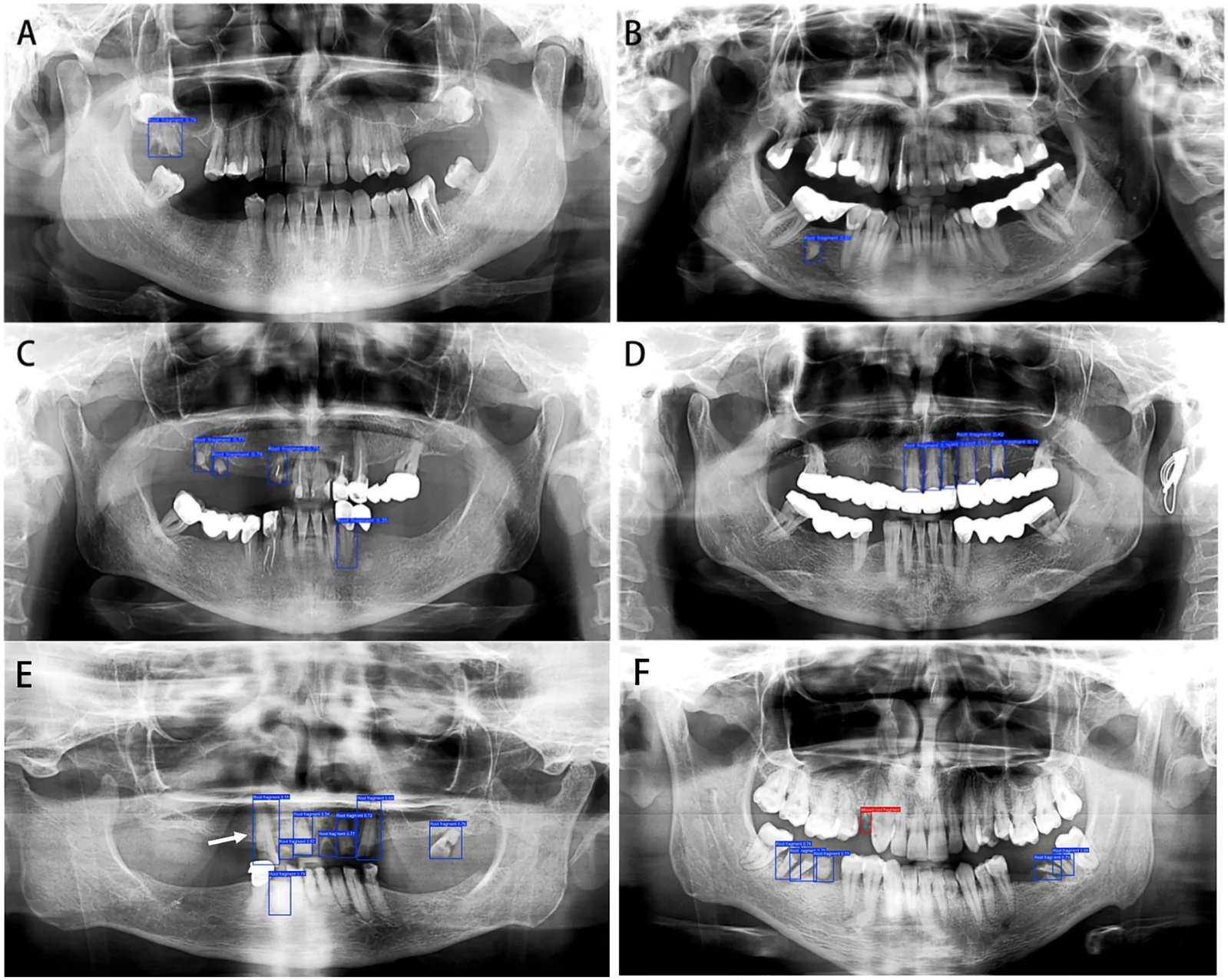
Detection results of YOLO12n model on panoramic images. The blue bounding boxes pinpoint detected root fragments across varying clinical presentations: (A): Decayed tooth root was successfully detected. (B): Successful identification of completely impacted or intraosseous root fragments within the alveolar bone. (C, D): Accurate detection of retained root fragments located in close proximity to or beneath existing coronal restorations (crowns and bridges), demonstrating the model's robust feature extraction in the presence of complex restorative materials. Panels (E, F) illustrate representative failure modes: (E) False positive (FP), where a severely worn/attrited tooth with loss of anatomical crown height was incorrectly classified as a retained root fragment due to morphological similarity; The red bounding boxes pinpoint missing detected root fragments: (F) False negative (FN), where a minute root fragment overlapping with the root of an adjacent intact tooth was missed due to anatomical superposition and low boundary contrast.

The marked systematic variation in optimal operating thresholds across models—ranging from 0.23 for YOLO12n to 0.47 for YOLOv8n—underscores the necessity of application-specific calibration rather than relying on default training configurations (e.g., confidence = 0.50). This discrepancy highlights the methodological value of distinguishing between epoch-level and threshold-level evaluations; post-hoc confidence threshold scanning unlocked additional performance, shifting YOLO12n’s mean F1 score from a best-epoch 0.885 to an optimal-threshold 0.888. This finding reinforces the recommendation that medical object detection tasks should implement post-training threshold calibration to rigorously optimize performance [31]. From a methodological perspective, threshold calibration allows investigators to tune the sensitivity–precision trade-off according to specific diagnostic priorities. For instance, operating at a lower confidence cutoff maximizes lesion sensitivity, whereas higher thresholds suppress spurious background detections. Future software implementations could conceptually incorporate adaptable threshold settings depending on task-specific tolerances for false positives, though defining optimal clinical thresholds will require formal human-reader studies.

Several limitations of the present study highlight critical avenues for future research. First, our single-class design does not reflect the multi-class reality of clinical environments; future frameworks must expand to concurrently detect diverse coexisting dental conditions. Second, while the models demonstrated high detection rates at standard overlap thresholds (mAP@0.5 > 0.89), the substantial drop at stricter thresholds (mAP@0.5:0.95 = 0.427–0.447) highlights limitations in precise spatial delineation. Fine root fragments often exhibit low contrast and taper into faint apices that blend into the surrounding trabecular bone or overlap with cortical landmarks (e.g., maxillary sinus cortical floor and mandibular canal). Consequently, while the current models demonstrate technical feasibility for lesion-level localization within this positive-only benchmark cohort, evaluating true screening effectiveness and false-alert rates across unselected, asymptomatic populations requires broader prospective investigations. Third, the absence of external validation limits generalizability, making cross-institutional validation an essential prerequisite for clinical translation [30]. Fourth, although nano-scale variants offer practical computational efficiency, they may underestimate the ultimate performance ceiling. Future investigations should thus evaluate the performance of larger model variants and explore ensemble strategies to further push the boundaries of diagnostic accuracy. Finally, the lack of subgroup analyses and formal human-expert comparisons limits the immediate assessment of real-world utility. Addressing these gaps will require prospective multi-reader, multi-case (MRMC) trials coupled with explainability methods, such as Grad-CAM, to rigorously evaluate diagnostic impact, workflow efficiency, and clinician trust. Furthermore, it must be noted that our dataset was constructed exclusively from radiographs containing at least one confirmed root fragment. Consequently, this study tests case-only lesion detection rather than general diagnostic screening in a healthy population. The reported FP/image rates therefore reflect the alert burden in positive cases; applying this model to completely negative routine screening populations could yield different false-positive dynamics.

## Conclusion

In conclusion, this study demonstrates the technical feasibility of nano-scale YOLO object detectors for identifying retained root fragments in dental panoramic radiographs within a controlled experimental setting. However, prior to considering clinical utility, extensive multi-center external validation and prospective clinical trials across unselected screening populations are required.
